# How Do Microorganisms Influence the Development of Endometriosis? Participation of Genital, Intestinal and Oral Microbiota in Metabolic Regulation and Immunopathogenesis of Endometriosis

**DOI:** 10.3390/ijms241310920

**Published:** 2023-06-30

**Authors:** Anna Sobstyl, Aleksandra Chałupnik, Paulina Mertowska, Ewelina Grywalska

**Affiliations:** Department of Experimental Immunology, Medical University of Lublin, Chodzki Street, 20-093 Lublin, Poland; sobstylanna.1@gmail.com (A.S.); olachalupnik@op.pl (A.C.); ewelina.grywalska@umlub.pl (E.G.)

**Keywords:** endometriosis, microbiota, dysbiosis, estrogen, estrobolome, metabolome, *Lactobacillus* spp., vaginal microbiome, uterine microflora, intestinal microflora, inflammation, immune dysregulation

## Abstract

Microorganisms inhabiting the human body play an extremely key role in its proper functioning, as well as in the development of the immune system, which, by maintaining the immune balance, allows you to enjoy health. Dysbiosis of the intestinal microbiota, or in the oral cavity or reproductive tract, understood as a change in the number and diversity of all microorganisms inhabiting them, may correlate with the development of many diseases, including endometriosis, as researchers have emphasized. Endometriosis is an inflammatory, estrogen-dependent gynecological condition defined by the growth of endometrial cells outside the uterine cavity. Deregulation of immune homeostasis resulting from microbiological disorders may generate chronic inflammation, thus creating an environment conducive to the increased adhesion and angiogenesis involved in the development of endometriosis. In addition, research in recent years has implicated bacterial contamination and immune activation, reduced gastrointestinal function by cytokines, altered estrogen metabolism and signaling, and abnormal progenitor and stem cell homeostasis, in the pathogenesis of endometriosis. The aim of this review was to present the influence of intestinal, oral and genital microbiota dysbiosis in the metabolic regulation and immunopathogenesis of endometriosis.

## 1. Introduction

Endometriosis is a chronic, inflammatory, estrogen-dependent gynecological condition defined by the growth of endometrial cells outside the uterine cavity [1]. Endometrial cells migrate from their original site, the uterus, to other organs and form endometrial-like tissues in various anatomical locations outside the uterine cavity, especially in the ovaries and peritoneum [1,2]. Although the symptoms of endometriosis are not unique and many of them are similar to the symptoms of other gynecological diseases, it can cause pelvic discomfort and infertility [3]. Literature data estimate that it is diagnosed with a significant delay of 3 to 11 years, which results in disruption of the reproductive cycle in women of reproductive age. The true prevalence of endometriosis cannot be determined due to the lack of effective non-invasive diagnostic techniques; nevertheless, epidemiological data indicate that about 10% of women of childbearing age suffer from endometriosis. Additionally, its incidence increases from 20% to 50% in women experiencing pelvic discomfort or infertility [3,4,5]. Endometriosis is a disease with a complex etiopathogenesis, which involves the participation of genetic and immunological pathways (including inflammation), as well as environmental influences, such as eating habits and nutrients, or antibiotic therapy, which, according to recent research, may be one of the factors modifying the amount of and diversity of microflora. Research indicates that the development of endometriosis involves alterations in multiple biological pathways, mainly the metabolic regulation at the basis of estrogen-dependent disorders [6,7]. The aim of this review was to present the influence of intestinal, oral and genital microbiota dysbiosis in the metabolic regulation and immunopathogenesis of endometriosis.

## 2. The Importance of Disorders of Immune Homeostasis in the Pathogenesis of Endometriosis

Endometriosis is a chronic inflammatory disease of immune origin characterized by the presence of endometrial-like tissue outside the uterus, commonly found in the pelvic cavity and other abdominal organs. The ectopic tissue exhibits similar characteristics to the endometrium, including the ability to respond to hormonal fluctuations and undergo cyclic shedding and bleeding [8]. Several mechanisms have been proposed for the histologic origins of endometriosis. One theory suggests that fragments of menstrual endometrium pass retrograde through the fallopian tubes and implant on peritoneal surfaces [9]. Another hypothesis is that the peritoneum undergoes metaplasia to form endometriotic lesions within the peritoneal cavity [10]. There are also suggestions that menstrual tissue reaches distant body sites via veins or lymphatic vessels [11], or that circulating blood cells differentiate into endometriotic tissue [12]. However, although various theories have been proposed which posit issues such as genetic predisposition, immune dysfunction and hormonal imbalances as contributing factors, the precise etiology of endometriosis remains unclear. Local inflammation, which plays a major role in endometriosis, causes pain and infertility, as well as affecting the development and progression of the disease [13]. Numerous studies have shown that the peritoneal fluid of patients with this disease contained large amounts of immune cells and macrophages that produce cytokines. What is more, in recent studies, oxidative stress, an imbalance between the production of reactive oxygen species (ROS) and the antioxidant defense system, has emerged as a potential contributor to the pathogenesis of endometriosis. Increased ROS production and reduced antioxidant capacity have been observed in the peritoneal fluid, serum and endometrial tissues of individuals with endometriosis [14,15,16]. Under physiological conditions, cytokines counteract the harmful effects of persistent or excessive inflammatory reactions that can have harmful effects. When the equilibrium between the creation and detoxification of reactive oxidative species (ROS) is interrupted, the relative excess of ROS causes oxidative stress. ROS are reactive oxygen-containing chemical species, such as superoxide (O_2_^−^), hydroxyl radicals (·OH) and hydrogen peroxide (H_2_O_2_). ROS in high concentrations can harm peritoneal mesothelial cells and induce ectopic endometrial implantation [17,18]. It can also alter the shape and function of vascular endothelial cells, such as permeability and adhesion molecule expression, hence increasing the adhesion between inflammatory cells and endothelial cells and leading to prolonged inflammation [19,20]. What is more, ROS can activate NF-κB and protein kinase B (AKT) pathways promoting endometriosis development [20,21]. In pathological conditions, the action of anti-inflammatory mediators may be insufficient or overcompensate for the inflammatory response, leading to inhibition of the immune response, which increases the risk of systemic infection [22]. Elevated concentrations of immune mediators, mainly pro-inflammatory molecules, occur not only locally but also systemically. In damaged endometrial cells, nuclear factor kappa-light-chain-enhancer of activated B cells (NF-κβ) is the major transcription factor that is overexpressed. The cytokine-mediated pro-inflammatory environment further enhances this effect. In turn, NF-kβ is involved in the positive regulation of these pro-inflammatory factors [22]. For many years, studies have shown that not only the participation of individual subpopulations of cells of the immune system, but also elevated levels of anti-inflammatory cytokines may be involved in the development of endometriosis.

### 2.1. The Role of Immune Cells in the Development of Endometriosis

#### 2.1.1. Macrophages

Macrophages can be divided into two groups: M1 macrophages, which have pro-inflammatory effects, and M2 macrophages, which have anti-inflammatory effects and can induce tissue remodeling through profibrotic effects. M2 macrophages also have proangiogenic properties and can induce immunotolerance [23]. An increased number of macrophages was observed in the peritoneal fluid and in the eutopic endometrium of women with endometriosis [24]. However, an increased number of these cells was not observed in peripheral blood in this group of patients [25,26]. Research results suggest that macrophages can directly affect endometrial cells. Co-culture of macrophages with these cells increased cell proliferation, increased invasiveness and led to the activation of the signal transducer and activator of transcription 3 (STAT3) in endometriotic stromal cells [27,28]. The current study demonstrates the ability of macrophages to polarize. Polarity is a spatiotemporally organized field that is time- and tissue-dependent. This phenomenon is associated with both internal, external and environmental tissue signals, including growth factors, cytokines, prostaglandins and pathogen-derived molecules. Polarization is evidenced by changes in phenotype such as its loss, switch or modulation. Depending on the disease state, macrophages show different polarization, which demonstrates their plasticity. In inflammatory diseases, an M1-like polarity is more frequently observed, whereas in chronic bacterial, parasitic or viral diseases, an M2-like polarity predominates [29].

#### 2.1.2. Neutrophils

The number of neutrophils in the peritoneal fluid of endometriosis patients may be increased [30]. When tested in a mouse model, it was observed that reducing the number of neutrophils with the anti-Gr-1 antibody resulted in a reduction in lesion formation. Neutrophils aggravate endometriosis mainly through the expression of pro-inflammatory cytokines such as interleukin 8 (IL-8 or CXCL8), C-X-C motif chemokine ligand 10 (CXCL10) and vascular endothelial growth factor (VEGF), which may promote disease progression [31,32]. One study observed that incubation of neutrophils from disease-free women with plasma or peritoneal fluid from women with endometriosis reduced the rate of apoptosis compared to control women. This study demonstrated the potential presence of apoptotic factors or PF in the plasma of women with endometriosis that are absent in healthy women [33].

#### 2.1.3. Natural Killer Cells

Natural killer (NK) cells are the primary cell population of the immune system and exhibit cytotoxic activity. They are also important for proper tissue homeostasis. NK cell counts appear to be the same in patients with and without endometriosis [24]. The endometrium in healthy women has an immunosuppressive effect on NK cell activity. Most likely, this will facilitate the implantation of the embryo. It has been observed that NK cells show even less cytotoxicity in the peritoneal fluid of endometriosis patients, which may contribute to the transformation of endometrial fragments into lesions [34]. In the natural killer cells in the peritoneal fluid of women with endometriosis, the cytotoxic activating killer-cell immunoglobulin-like receptor (KIR) is inhibited as a result of upregulation of IR2DL1, which may contribute to lower cytotoxicity of these cells [35]. Another mechanism responsible for the reduced cytotoxicity of NK cells may be the expression of TGFB1, which inhibits the expression of NKG2D ligands [36]. The results indicate that IL-6 may be responsible for the immunosuppressive effect against NK cell cytotoxicity to autologous endometrial fragments [37].

#### 2.1.4. T Cells

CD4+ T cells can be subdivided into Th1 cells, which secrete interferon gamma (IFN-γ), and Th2 cells, which secrete IL-4 [38]. In addition, Th17 lymphocytes and regulatory T lymphocytes (Treg) are distinguished [39,40]. Women with endometriosis have been observed to have more Th2 lymphocytes as a result of strong intracellular expression of IL-4 and lack of IL-2 in lymphocytes isolated from ectopic lesions [41]. Th2 cells, through the secretion of IL-4 and IL-13, which mediate the differentiation of resident fibroblasts and the recruitment of fibrocytes to myofibroblasts, may contribute to pelvic fibrosis in women with endometriosis [42]. Studies show that in the case of ectopic endometriosis, a greater number of Th17 cells are present in the peritoneal fluid and peripheral blood compared to women without the disease [43]. Th17 cells express CCR6 (C-C Motif Chemokine Receptor 6), a receptor for the CCL20 (C-C Motif Chemokine Ligand 20) ligand that is produced by endometriosis stromal cells and has a chemotactic effect on Th17 cells [44]. Treg lymphocytes are also more numerous in ectopic lesions. The results of the study showed that in the peritoneal fluid and peripheral blood of patients with endometriosis, their concentrations were higher than in the control group [45,46]. A positive correlation was also found between the number of these cells and disease progression [47].

#### 2.1.5. Tight Junctions

Tight junctions are responsible for regulating and restricting transport between cells, thereby forming a selective barrier between them. They also act as fences by maintaining a tight organization of the plasma membrane of epithelial cells in the apical and basolateral compartments [48]. Tight junctions are mainly composed of claudins, which are found in the apicolateral membranes of epithelial and endothelial cells [49]. Based on function, claudins can be divided into channel-forming (claudin-2, -4, -10, -15, -17 and -21) and barrier-forming (claudin-1, -3, -5, -11, -14 and -18) [50]. Several claudins such as claudin-1, -5, -7 and -11 have been identified in the human endometrium [51,52]. Studies have shown lower expression of claudin-3 and claudin-4 in ectopic endometriosis tissue compared to eutopic endometrium in women with endometriosis at messenger mRNA and protein levels. These changes may play a role in the pathogenesis of this disease [53]. On the other hand, a study by Hoerscher et al. showed that claudin-2 and claudin-3 show similarity in localization in the ectopic compared to the eutopic endometrium [54]. In addition, Gaetje et al. showed that there is reduced expression of claudins in peritoneal endometriosis lesions in comparison with eutopic endometrium. Downregulation of claudin-3 and claudin-7 in endometriotic lesions and reduction of claudin-5 mRNA were observed [51].

#### 2.1.6. Damage-Associated Molecular Patterns (DAMPs) and Pathogen-Associated Molecular Patterns (PAMPs)

Endometriosis is linked to ectopic localized inflammation and an immunocompromised microenvironment. The innate immune system has a variety of pattern recognition receptors (PRRs) that can detect danger-associated molecular patterns (DAMPs) and pathogen-associated molecular patterns (PAMPs) in both intracellular and extracellular environments. Danger-associated molecular patterns reflect host tissue damage. These include heat shock protein 70 (HSP70), adenosine-5′-triphosphate (ATP), high-mobility group box protein 1 (HMGB1) and IL-33. They are released after cell death during inflammation. It has been shown that some DAMPS such as DNA, ATP, HMGB1 and HSP70 are involved in endometriosis [55]. HMGB1 is involved in necrosis and severe cellular stress [56]. It is expressed in epithelial and stroma cells in both endometriosis patients and disease-free patients [57]. Kajihara et al. showed that DAMPs promote the development and progression of endometriosis in association with TLR activation and NF-κB signaling. Their work also suggested that histones may be a new class of DAMP molecules and activate innate immunity during sterile inflammation in endometriosis [55]. The findings suggest that inhibitors of DAMP molecules may represent a potential therapeutic target for preventing or reducing the progression of endometriosis. This is possibly due to the function of these molecules as a link between infection-induced initial cellular damage and activation of innate immune cells as a result of oxidative stress and sterile inflammation [58].

PAMPs have the ability to differentiate between harmful microorganisms and the host’s own cells [59,60,61,62,63,64]. PAMPs are structures found in various microorganisms [65], such as bacterial lipopolysaccharide, lipoprotein, peptidase, flagellin, unmethylated CpG dinucleotide motifs (CpG-DNA), viral double-stranded RNA, fungal cell wall, and others. Upon recognizing PAMPs, PRRs initiate a reaction between the receptor and the ligand, transmitting signals of microbial infection to the cells. This process stimulates the immune response of the host, leading to the elimination of the pathogenic microorganisms [66].

#### 2.1.7. Toll-Like Receptors (TLRs)

Based on the homology of their protein domains, PRRs can be categorized into five groups. These groups include toll-like receptors (TLRs), c-type lectin receptors (CLRs), nod-like receptors (NLRs), retinoic acid-inducible gene I-like receptors (RLRs) and absent in melanoma 2 (AIM2)-like receptors (ALRs). These receptor groups can be further classified into two main classes: membrane-bound receptors and intracellular receptors [67]. The first class primarily consists of TLRs, which are typically found on the cell membrane but may occasionally be located within intracellular compartments (e.g., TLR3). TLR activation triggers a series of events that leads to the transcription of many downstream genes involved in inflammatory response and antimicrobial defense [68]. TLR4, TLR3 and TLR2 are members of the TLR family, mostly found in samples studied for endometriosis. TLR4 is expressed by various immune cells and has been found to be higher in endometrial stromal cells of endometriosis patients. Animal experiments have demonstrated that activation of the LPS/TLR4 pathway, induced by injecting lipopolysaccharide (LPS) into a mouse model of endometriosis, leads to the release of inflammatory factors and promotes the proliferation and invasion of ectopic endometrial stromal cells [69]. TLR3 is involved in innate immunity and its activation induces the production of inflammatory cytokines. TLR3 expression is increased in both ectopic and eutopic endometrium of endometriosis patients, contributing to the inflammatory state and enhanced cell viability [70]. TLR2 plays a role in bacterial infections and the innate immune system. Its expression has been assessed in endometriosis patients, but further research is needed to understand its specific involvement [71]. Overall, more studies are needed to explore the presence and potential use of TLRs as biomarkers for the early detection of endometriosis.

#### 2.1.8. Lipopolysaccharide

Local and chronic inflammatory reactions in the peritoneal environment play a key role in the pathogenesis of endometriosis. The menstrual blood of women with endometriosis contains a higher concentration of lipopolysaccharide (LPS) compared to women without the disease [72]. Lipopolysaccharide is an inflammatory mediator that is a component of Gram-negative bacterial cells. Under its influence, peritoneal macrophages secrete a variety of compounds such as vascular endothelial cell growth factor (VEGF), interleukin (IL)-6, IL-8, hepatocyte growth factor (HGF) and tumor necrosis factor-alpha (TNF-α) [58,73,74,75]. This LPS-induced secretion effect can be inhibited by the use of neutralizing antibodies for TLR4 and by polymyxin B, which is an LPS antagonist [72]. Khan et al. showed that high LPS content in peritoneal fluid (PF) due to menstrual blood reflux is associated with pelvic inflammatory disease and may contribute to TLR4-mediated progression of endometriosis [69]. Additionally, a study in mice showed that LPS, by generating peritoneal inflammation through activation of the TLR4/NF-κB pathway, exacerbated the development of endometriosis-like lesions [76].

### 2.2. The Importance of Inflammatory Mediators in Body Fluids (Peripheral Blood, Peritoneal Fluid, Urine) in the Development of Endometriosis

#### 2.2.1. Anti-Inflammatory Cytokines

IL-4 and IL-10 are the main anti-inflammatory cytokines involved in disease progression by stimulating the survival, growth, invasion and immune escape of endometrial lesions [41]. The results of several studies indicate their elevated levels in the lymphocytes and ectopic endometrium of patients with endometriosis [77,78,79]. Particularly high levels of these cytokines were observed in women with advanced endometriosis [80,81,82]. One study showed significantly higher levels of IL-4 in serum and peritoneal fluid in adolescents with endometriosis, which may be used in future work on the development of a biomarker of this disease [80]. A study in the Chinese population showed that the presence of polymorphisms 592A/C and 819T/C of the IL-10 promoter is associated with an increased risk of endometriosis and may be associated with increased disease progression [82,83,84].

Elevated concentrations of these cytokines may result from insufficient control of their pro-inflammatory effects, excessive compensation and inhibition of the immune response. IL-4 is abundant in endometrial tissues and causes activation of p38 mitogen-activated protein kinases, which are a class of mitogen-activated protein kinases (MAPKs), stress-activated protein kinases (SAPK)/Jun amino-terminal kinases (JNK) and p42/44 MAPK signaling, leading to the proliferation of endometriotic stromal cells [85]. The role of IL-10 in endometriosis has been studied in an animal model. Decreasing IL-10 activity in mice with surgically induced endometriosis significantly reduced the size of endometrial lesions. In contrast, IL-10 administration increased endometrial changes in this model [86]. Another anti-inflammatory cytokine is IL-13. Its increased concentration has been demonstrated in the endometrium and in the peritoneal fluid of patients with endometriosis [87]. However, elevated serum levels of it have not been demonstrated. The group of anti-inflammatory cytokines also includes transforming growth factor β1 (TGF-β), which inhibits or reverses the activation of macrophages, thereby reducing the release of pro-inflammatory cytokines and reactive oxygen and nitrogen species [88]. Animal model studies have shown that elevated levels of this cytokine are associated with increased survival, adhesion and proliferation of ectopic endometrial cells during lesion development in endometriosis patients [89,90]. In addition, studies conducted among patients with endometriosis showed an increased level of TGF-β in their peripheral blood [91].

#### 2.2.2. Pro-Inflammatory Cytokines

Studies indicate that elevated levels of pro-inflammatory cytokines can also be observed in the blood serum of women with endometriosis. A study by Borrelli et al. showed elevated levels of IL-8 and monocyte chemoattractant protein-1 (MCP-1/CCL2) in the peripheral blood of patients [92]. A study by Pizzo et al. also showed elevated IL-6 levels in this group of patients [86]. Elevated levels of another pro-inflammatory cytokine, IL-6, in peripheral blood were more common in patients with stage I-II endometriosis [86]. Tumor necrosis factor alpha (TNF-α) also belongs to the group of pro-inflammatory cytokines. Several studies have shown that its serum concentration in patients with endometriosis may be elevated [93]. The development and progression of endometriosis is also influenced by interactions between the two groups of cytokines. The pro-inflammatory cytokine IL-1β increases the secretion of anti-inflammatory IL-4 [94]. The pro-inflammatory cytokine TNF-α and the anti-inflammatory cytokine TGF-β1 significantly increase IL-13 secretion from endometrial epithelial cells (EECs) and endometrial stromal cells (ESCs) in vitro [95]. In contrast, IL-8 and IL-23 produced from ectopic lesions stimulate the differentiation of CD16—NK cells with high levels of cyclooxygenase (COX)-2, which express high levels of anti-inflammatory IL-10 and TGF-β1 [96].

#### 2.2.3. Urinary Markers of Inflammation and Endometriosis

The examination of endometriosis-specific changes in body fluids mainly involves peripheral blood and peritoneal fluid. However, more and more research is focusing on the search for non-invasive, disease-specific markers in the urine. A study by Visnic et al. [97] noticed as many as 17 proteins that were excreted in the urine of endometriosis patients and were involved in the pathogenesis of the disease. Proteins that were elevated in urine included ENG (endoglin), LUM (lumican), TGFB2 (transforming growth factor beta receptor 2), TSPAN1 (tetraspanin-1), CD44, TNC (tenascin), CatG (cathepsin G), DSP (desmoplakin), THBS1 (thrombospondin 1), PCDH1 and PDCH3, (protocadherin-1 and-3), SPARCL1 (SPARC-like protein 1), ZGP1 (zinc-alpha-2-glycoprotein) and ANXA2 (annexin A2) (see Table 1).

These proteins are responsible for cell invasion, proliferation and adhesion, neoangiogenesis, proteolysis and degradation of the extracellular space, processes playing an important role in the pathogenesis of endometriosis [129]. In 2021, a study was conducted evaluating the use of BCL6 (B-cell lymphoma 6) and SIRT11 (NAD-dependent protein deacetylase sirtuin-1) as markers in non-invasive diagnostics of endometriosis, but the results showed that the possibilities of their use are low [130]. Identifying reliable protein markers in urine could potentially provide a non-invasive method for diagnosing and monitoring endometriosis. Several studies have explored the identification of specific protein biomarkers in urine samples from women with endometriosis. These biomarkers are typically proteins that are released or altered in response to the presence of endometriotic lesions. The interaction between protein markers in urine and the microbiota in endometriosis is an area of ongoing research and is not yet fully understood. However, there are a few potential mechanisms by which the microbiota and protein markers in urine may interact in the context of endometriosis: inflammatory response, metabolic alterations and barrier function [131,132]. The presence of endometriotic lesions can trigger an inflammatory response in the reproductive tract, which may lead to changes in the microbiota composition. This altered microbiota, in turn, can contribute to the release of specific proteins into the urine. The inflammatory response and changes in the microbiota may act synergistically, further perpetuating the inflammatory state associated with endometriosis. Dysbiosis or alterations in the microbiota composition observed in endometriosis could influence immune dysregulation, allowing the development and persistence of endometriotic lesions. This immune dysregulation may also affect the expression or release of certain proteins into the urine. Dysbiosis in endometriosis may result in the production of specific metabolites that can impact protein expression or function, leading to their appearance in urine samples. The microbiota plays a role in maintaining the integrity of the epithelial barrier in the reproductive tract. Disruptions in this barrier function could allow the translocation of microbial components or proteins into the urine, potentially leading to the detection of specific protein markers associated with endometriosis [131,132].

A study aimed at examining selected adhesion molecules as urinary biomarkers for diagnosing endometriosis showed no differences in the levels of sVCAM-1, sICAM-1, E-selectin and P-selectin in the urine of patients with endometriosis compared to healthy patients [133]. Studies have also shown that CYFRA 21-1 in urine can be a valuable marker in the diagnosis of endometriosis [133,134]. Chen et al. observed significantly elevated urine histone 4 levels in patients with endometriosis [135]. Elevated levels of soluble fms-like tyrosine kinase (sFlt-1) corrected for creatinine have also been detected in the urine of patients with stage I and II endometriosis [133]. The use of cytokeratin-19 (CK19) as a biomarker was also investigated; however, research results are contradictory. Kuessel et al. showed that there is no significant relationship between urinary CK19 levels and endometriosis [136]. In turn, a study by Tokushige et al. showed that CK19 was expressed only in the urine of women with endometriosis and was absent in the urine samples of healthy patients [137].

Metabolites involved in inflammation and oxidative stress were also found in the urine samples of endometriosis patients. These samples contained higher levels of guanidinosuccinate, creatinine, taurine, valine, 2-hydroxyisovalerate and N-methyl-4-pyridone-5-carboxamide, and decreased lysine levels, compared to disease-free women [138]. An animal-model study showed that the tryptophan metabolites 4,6-dihydroxyquinoline and 5-hydroxy-L-tryptophan were significantly altered in the urine of model endometriosis rats [139]. Regarding estrogen metabolites, a study by Othman et al. found that the urine of women with endometriosis had significantly higher levels of 2-hydroxyestrone than women without the disease, but the ratios of 2-hydroxyestrone/16α-hydroxyestrone were comparable [140].

In 2021, a study was conducted to determine the association of microbial dynamics with estrogens and estrogen metabolites in the urine of endometriosis patients. The results showed that the concentrations of 17β-estradiol and 16-keto-17β-estradiol were higher in these patients. In addition, lower levels of estriol, 2-hydroxyestrone and 2-hydroxyestradiol were observed, mainly in patients using oral contraceptives. Another aim of the study was to analyze the relationship between the species of urinary bacteria and the concentration of estrogens in the urine. In a control group consisting of patients without endometriosis, an association was observed between bacteria in the urine and the concentration of 2-hydroxyestrone in urine samples. In patients with endometriosis, a strong correlation was found between urinary bacteria and the levels of E2, E3, 2-hydroxyestradiol and 2-hydroxyestrone. These results indicate that there is a relationship between abnormal levels of estrogens and their metabolites in the urine and altered composition of the urinary microbiome in patients with endometriosis. These findings suggest that patients with endometriosis may have a unique profile of estrogen metabolites [141]. 

Metabolic and hormonal disorders can have a significant impact on the microbiota in the context of endometriosis. The microbiota is highly influenced by the host’s metabolism, and changes in nutrient availability can promote the growth of certain bacterial species while suppressing others. This disruption in the metabolic environment can potentially lead to dysbiosis in the reproductive tract microbiota. Hormonal imbalances, particularly concerning estrogen, are not only a hallmark of endometriosis, but can also directly affect the growth of certain bacteria and alter the gene expression of microbes involved in metabolism and virulence. Therefore, endometriosis-related hormonal imbalances have the potential to disrupt the microflora of the reproductive system. Moreover, metabolic and hormonal disturbances in endometriosis may contribute to immune dysregulation that affects the diversity and composition of the microflora and the integrity of the mucosal barrier in the reproductive system. Disruption of barrier function can allow translocation of microbial components and metabolites into surrounding tissues, leading to inflammation and immune activation. This altered barrier function may also affect the composition and diversity of the microflora [131,142,143,144].

## 3. Influence of Microbiota Dysbiosis of the Reproductive Tract, Intestines and Oral Cavity on the Pathogenesis of Endometriosis

### 3.1. The Microbiota of the Genital Tract and Endometriosis

Microorganisms and hosts have synergistic links in virtually every niche of the human body, affecting its physiology and physiopathology [145,146,147,148,149]. Similarly, the female genital organs (FGT)—the vagina, cervix, endometrium, fallopian tubes and ovaries—have their own microbiome, accounting for 9% of the total number of bacteria in a woman’s body [150]. Recent molecular studies have shown that the presence of bacteria in the endometrium plays an important role in the proper functioning of the endometrium and the development of pregnancy under normal conditions [151].

**Figure 1:**
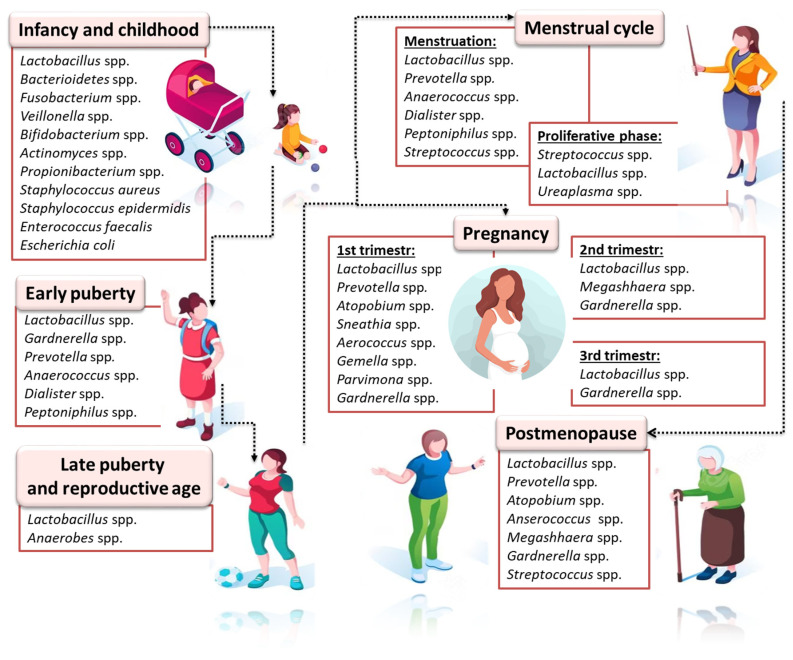
Microbiological differentiation of female reproductive tracts in various physiological conditions; based on [160,161,162]. The age frames of girls in particular stages of development are an individual feature; however, in order to standardize the changes in the composition and number of microorganisms of the reproductive tract, let us assume that the period of infancy and childhood applies to girls up to the age of 8; early puberty is 8–13 years old; late puberty is 14–17 years old; reproductive age is 18–40 years; and postmenopause covers women over the age of 55. In addition, in the diagram, the microorganisms are listed in descending order of predominance.

The vaginal microbiome tends to play a significant role in the prevention of urogenital diseases such as bacterial vaginosis, inflammatory diseases, sexually transmitted infections and urinary tract infections. This protective role is primarily due to *Lactobacillus* spp., which is often associated with good gynecological and reproductive health. *Lactobacillus* spp. produce lactic acid and various bacteriostatic and bactericidal components that contribute to lowering the pH of the vaginal environment to a value less than or equal to 4.5 and promote competitive exclusion [152]. The estrogen-dominant vaginal epithelium and host amylase are essential for the survival and growth of *Lactobacillus* spp. [153,154,155]. In addition, *Lactobacillus* spp. help maintain homeostasis by occupying this niche (pathogen exclusion) and producing anti-inflammatory cytokines and antimicrobial peptides from epithelial cells, which strengthens the epithelial cell barrier and gynecological health [156,157,158,159]. The composition and number of microorganisms is extremely diverse in different physiological (menstruation, menopause) (Figure 1) and pathological conditions.

Several studies have proven that *Lactobacillus* is the dominant genus in the endometrium as well as in the vaginal environment. Mitchell et al. studied the microflora of the vagina and uterus and found that the endometrium is distinguished by the presence of *Lactobacillus*, the most widespread genus, followed by *Gardnerella*, *Prevotella*, *Atopobium* and *Sneathia* [163]. However, the composition of the vaginal microflora varies at different stages of menopause (pre-menopausal, perimenopausal and post-menopausal), as well as in pathological conditions such as vaginal atrophy, when the number of *Lactobacillus* decreases and the number of microorganisms such as *Anaerococcus*, *Peptoniphilus* and *Prevotella* increases [164]. Further research by Verstraelen et al. showed that 90% of the results included in their study were consistent with the presence of a unique microflora dominated by Bacteroides (*Bacteroides xylanisolvens*, *Bacteroides thetaiotaomicron* and *Bacteroides fragilis*) and *Pelomonas* residing on the endometrium of the human non-pregnant uterus [165].

Research conducted by Chen’s team indicates the presence of many bacterial communities, the most numerous of which are *Pseudomonas* spp., *Acinetobacter* spp., *Vagococcus* spp. and *Sphingobium* spp. [145]. Similar results were obtained by sequencing endometrial samples from 25 women who underwent total hysterectomy for fibroids or endometrial hyperplasia and it was found that *Acinetobacter* spp., *Pseudomonas* spp., *Comamonadaceae* spp. and *Cloacibacterium* spp. were the most prevalent taxa in the endometrium [166]. Recent research by the team of Lu et al. in 2021 showed that *Rhodococcus* spp., *Phyllobacterium* spp., *Sphingomonas* spp., *Bacteroides* spp. and *Bifidobacterium* spp. were more numerous in the endometrium than *Lactobacillus* spp. in patients diagnosed with endometrial cancer [167]. Until now, the lack of contamination control, evidence of bacterial viability and transcervical sampling has made it difficult to evaluate the results of these genital tract microbiota studies and their direct impact on the pathogenesis of endometriosis.

In recent years, some scientists have proven the non-sterility of the endometrium. There is evidence that bacteria can enter the uterus as “tourists” or “invaders”, as discussed in a study by Baker et al. (2018). Furthermore, embryos from a woman’s lower genital tract can be transferred in and out of the uterus by the peristaltic pump of the uterus [168], and the peristaltic contractions of the uterine fundus rapidly transport sperm out of the canal cervix to uterus and fallopian tube [169]. The frequency of these contractions changes as the proliferative phase progresses, as was observed using labeled macrospheres over several minutes [169]. In women with infertility and endometriosis, uterine hyperperistalsis has been documented with approximately twice the frequency of contractions [170]. This hyperperistalsis in patients with endometriosis may contribute not only to the transfer of detached endometrial cells to the fallopian tubes and peritoneal cavity, but also to the transfer of microorganisms from the lower genital tract. Mid-cycle uterine dysperistalsis can occur in women, causing undirected and convulsive contractions that can lead to infertility by interfering with sperm transport, but can also result in a high probability of translocation of bacteria into the upper vaginal canal. Several studies have reported some abnormalities of the endometrium in the predominant types of patients with endometriosis. Indeed, Fang et al. compared the bacterial composition of the vagina with the endometrium, as well as the composition of the endometrial microbiome of healthy women, patients with endometrial polyps and patients with chronic endometritis. *Proteobacteria* spp., *Firmicutes* spp. and *Actinobacteria* spp. dominated the intrauterine microbiome in all groups studied. Additionally, although there were significant differences in the vaginal and endometrial microbiome, numbers of *Lactobacillus* spp., *Gardnerella* spp., *Bifidobacterium* spp., *Streptococcus* spp. and *Alteromonas* spp. were significantly higher in the healthy group compared to others [171]. Ata et al. conducted a study to compare the composition of the vaginal, cervical and intestinal microflora of women with stage III/IV endometriosis with healthy controls. A difference was identified at the genus level. They found an increased abundance of potentially pathogenic species in the cervical microbiota of women with endometriosis, including *Gardnerella* spp., *Streptococcus* spp., *Escherichia* spp., *Shigella* spp. and *Ureaplasma* spp. Surprisingly, they did not find *Atopobium* spp., a gynecological pathogen, in the vagina and cervix of the endometriosis group [172]. Another study showed a significant incidence of *Atopobium vaginae* in women. *Atopobium* may exacerbate intracellular *Porphyromonas* infection, causing changes in cell regulatory processes and a carcinogenic trigger in women with endometrial cancer. In contrast, they found that *A. vaginae* was less common in women with mild gynecological disorders, suggesting a possible link at a different stage of action, given that endometriosis is also a benign gynecological disease [173].

**Figure 2:**
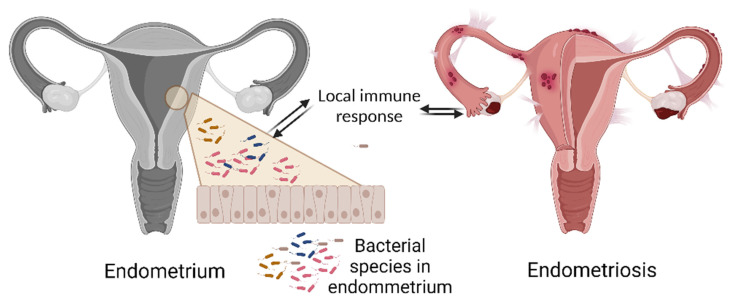
The interaction of bacteria with the endometrial epithelium. Changes in the vaginal and intestinal diversity of the microbiome resulted in an immunological shift towards an inflammatory profile, which may promote inflammation by producing inflammatory mediators; based on [174].

Since it is still unknown whether these changes are the cause or effect of the disease, studies in non-human primates (*Papio anubis*) were conducted to test the hypothesis that the development of endometrial changes causes changes in immune and bacterial dynamics that may promote disease progression. According to the study, the induction of endometriosis decreased peripheral Tregs while increasing the number of Th17 cells in all post-induction harvests, indicating systemic inflammation. After disease induction, the diversity and abundance of the microbiome changed in the vaginal and intestinal microbiota. Consequently, the induction of endometriosis in primates resulted in an immunological shift towards an inflammatory profile, as well as altered mucosal microbial profiles, which may promote inflammation by producing inflammatory mediators [174] (Figure 2).

The vaginal community status type (CST) consists of five CSTs. CST I, II, III and V are associated with a healthy vaginal microbiome and are dominated by *Lactobacillus gasseri*, *Lactobacillus crispatus*, *Lactobacillus iners* and *Lactobacillus jensenii*. CST IV is associated with vaginal inflammation and dysbiosis. This type consists of more strictly anaerobic bacteria (e.g., *Dialister*, *Gardenerella*, *Megasphaera*, *Peptoniphilus*, *Sneathia*, *Finegoldia* and *Prevotella*) [160]. The presence of specific species of bacteria in the urogenital (UG) system, such as *Lactobacillus* spp. or *Bifidobacterium infantis*, protects mucosal epithelial cells and creates an environment that prevents pathogen survival by producing lactic acid, which lowers vaginal pH [175].

In 2022, Le et al. conducted a study on reproductive olive baboons to assess the impact of endometriosis on mucosal microbial community immunity and dynamics. Comparison of pre-disease samples with samples from baboons with endometriosis showed that disease induction changed the dynamics of the urinary microbiome. As the disease progressed, the levels of a large proportion of species in the urinary tract decreased. The effect of lower diversity of microorganisms due to dysbiosis and inflammation is lower metabolic activity of microorganisms, which can lead to the destabilization of immune homeostasis. This study also analyzed the effect of microbiome dynamics on immune status to determine whether the change in microbiome diversity following endometriosis induction was related to populations of peripheral immune cells. The results showed that urinary dysbiosis in affected animals is accompanied by changes in the levels of peripheral nTregs, iTregs and Th17 cell populations, suggesting that there may be a unique composition of the urinary microbiome as well as a distinct immune profile associated with the induction of endometriosis [173]. Sex hormones contribute to changes in the microbiome of the urinary tract. Decreased estrogen levels during menopause may result in a reduction in the number of commensal species [176]. In patients with endometriosis, an increase in the level of circulating bioactive estrogens may initiate the development and persistence of endometrial changes. Therefore, changes in the composition of the microbiome in these patients may affect estrogen production [177].

### 3.2. Gut Microbiota and Endometriosis

The digestive tract is heavily filled with lymphoid structures that contain cells associated with the immune system [178]. It is well known that the intestinal microflora plays an important role in the development of these structures, as well as in the development of immune cell activity. In fact, animal germ-free models have shown that the absence of gut microbiota is correlated with deficiency in secretory IgA and CD8αβ intraepithelial lymphocytes [178]. The gut microbiota also affects the composition of mucosal T lymphocytes (Th1, Th17, Treg, etc.) and dysbiosis can disrupt this delicate balance, causing inflammation and various diseases. These investigations proved that the gut microbiota has a putative role in the function of immune system cells [178]. In addition, commensal microorganisms compete for resources by limiting the colonization of harmful microbes. For example, lactobacilli inhibit the binding of *Neisseria gonorrhoeae* to the female reproductive system, protecting the host from infection [179]. Commensal bacteria also constantly excite the receptors, resulting in an increase in the number of TLRs and, as a result, enhanced immune surveillance [180]. While certain types of gut bacteria contribute to the development of endometriosis, other microorganisms also help create a healthy barrier in the gut. The impact of gut bacteria on host physiology and immunological processes is mediated through various mechanisms. One such mechanism involves the breakdown of otherwise indigestible nutrients into biologically active metabolites, including short-chain fatty acids (SCFAs) [181,182]. SCFAs such as acetate, propionate, n-butyrate, pentanoic acid (valeric acid) and hexanoic acid (caproic acid) serve as an energy source for enterocytes or are transported into the bloodstream [183]. These SCFAs have been shown to exhibit anti-proliferative effects [184,185,186] and possess anti-inflammatory properties [187] that can extend to distant organs [188]. In lipopolysaccharide-induced macrophages, n-butyrate inhibits the expression of pro-inflammatory cytokines such as tumor necrosis factor α (TNF-α) and IL-6 [189]. SCFAs primarily exert their effects on cells through two main mechanisms. Firstly, they can activate G-protein-coupled receptors, namely, GPR43, GPR41 and GPR109A [190], which are known to downregulate inflammation [191,192]. Secondly, SCFAs can inhibit histone deacetylases [193,194]. Furthermore, it is observed that the fecal matter of mice with endometriosis contained lower levels of short-chain fatty acids and n-butyrate compared to mice without the condition. In a pre-clinical mouse model, treatment with n-butyrate resulted in a decrease in the growth of both mouse and human endometriotic lesions [195].

In addition, microorganisms play an important role in the physiological functions of other mucosal surfaces, such as endometrial remodeling in the uterus and increasing estrogen levels in the blood [180]. Recent studies have shown that the unique EMS microenvironment may favor the propensity of M2 endometrial macrophages to polarize towards M1 polarization and that M2 macrophages paradoxically express pro-inflammatory phenotypes in the early phase, although the pro-inflammatory phenotype of M1 macrophages is promoted when endometrial changes are established [196].

A significant number of Gram-negative bacteria are translocated and infiltrated outside the intestinal cavity as a result of the altered composition of the intestinal microbiota induced by endometriosis, which results in the destruction of intestinal tight junctions and the reduction of tight junction protein 2 (ZO-2) expression [197], resulting in the infiltration of a significant amount of Gram-negative bacteria outside the intestine [198].

According to Harada et al., LPS can activate the macrophage TLR4 in innate immunity, leading to the production of significant levels of TNF-α and IL-8 and the development of an inflammatory environment [199]. TNF-alpha and IL-8 are crucial for endometrial tissue adhesion and angiogenesis induction [200]. The expression of pro-inflammatory phenotypes by M1 macrophages may be facilitated by activation of TLR4 signaling and the inflammatory environment [201]. Firmicutes, Bacteroidetes, Proteobacteria, Actinobacteria, Spirochaetes and Fusobacteria phyla predominate in the population of oral bacteria, and the main properties of these major bacterial taxa have been previously characterized [202,203]. The list of bacterial genera and types in the publicly available Human Oral Microbiota Database (HOMD) is constantly updated. The HOMD study showed that each person has a distinct microflora. The “true” oral microflora mix is difficult to identify because the oral cavity is constantly exposed to the external environment, allowing exogenous microorganisms, air and food to enter [203]. It should be noted that bacterial populations may vary depending on the oral sampling site. Differences in oral microbial diversity have been identified at various sites in the oral cavity, particularly between the mucosa and teeth and between saliva and teeth [204]. A recent study showed that the microbiota of the buccal, gingival and hard palate mucosa were comparable, although saliva, tongue, tonsils and pharynx as well as supragingival and subgingival plaques had altered taxa [205]. So far, saliva is the most perfect oral compartment to study changes in microbial composition in many human diseases, because it contains the most representative oral microflora [206]. The oral microbiome can significantly contribute to the development of various human diseases, most of which are caused by the accumulation of oral bacteria leading to inflammation [207].

Although little is known about the oral microbiome and endometriosis, other inflammatory conditions can affect the oral microbiome. While inflammatory bowel disease (IBD) is a chronic inflammatory condition that affects the digestive tract, patients with IBD can develop oral mucositis [208]. Statistically significant differences in the oral microflora of young IBD patients have been shown to be significantly less than in healthy controls, with a significant reduction in Fusobacteria and Firmicutes phyla [209]. Behçet’s syndrome is a multi-immune systemic disease characterized by an enhanced systemic inflammatory response. Mouth ulceration is a common symptom of this condition. The microbiome of the oral mucosa and saliva of patients with Behçet’s syndrome differed from that of healthy individuals; a significant proportion of *Actinobacteria* spp. were found in patients with Behçet’s disease and oral ulcers [210]. The extremely inflammatory nature of this condition may affect the composition of the oral microbiome. Dysfunction of the oral microbiome is also associated with other chronic inflammatory diseases such as atherosclerosis and cardiovascular problems. Slocum et al. analyzed the putative pathways through which periodontal infections directly or indirectly stimulate immune dysregulation and, as a result, the progressive inflammation that manifests in cardiovascular disorders [211]. Furthermore, autoimmune diseases have the potential to disrupt the host’s commensal oral microbiota. Rheumatoid arthritis (RA) is an inflammatory disease mainly characterized by inflammation of the joints. The oral and salivary microbiota of RA patients has been shown to be significantly different from that of healthy humans using 16S rRNA sequencing, which closely matches clinical RA measurements such as C-reactive protein levels [212]. The chronic inflammatory nature of RA may play a significant role in the dysbiosis of the oral flora, allowing oral pathogens to colonize the oral region. As a consequence, oral dysbiosis causes changes in the relative abundances of individual components of the bacterial community. Immunological identification of periodontal infection causes an increase in inflammation, which includes the influx of immune cells such as monocytes, B and T lymphocytes, and the synthesis of inflammatory mediators [213,214]. When inflammation becomes persistent, it can contribute to endometriosis. Substantial long-term studies are required to confirm the current evidence implicating oral dysbiosis as an independent risk factor for endometriosis.

## 4. Metabolic Regulation of Estrogen Metabolism and Microbiota

**Figure 3:**
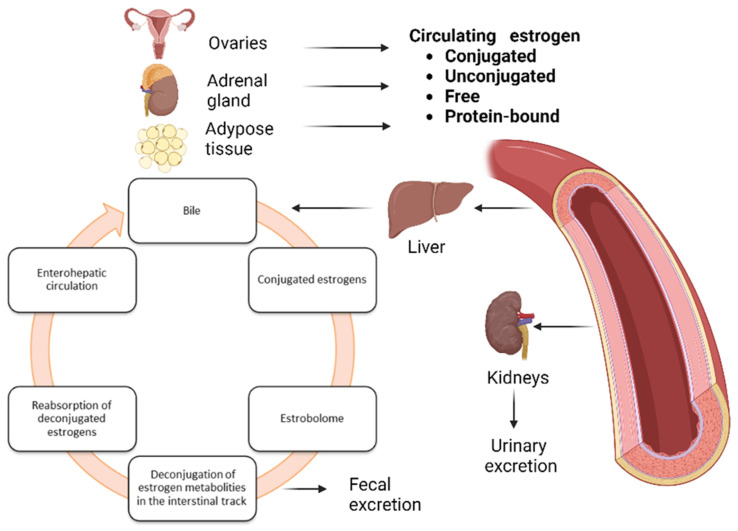
Metabolism of estrogen in the human body; based on [224,225].

As mentioned earlier, estrogens are essential for the development and functioning of the female reproductive system and are closely involved in the development of endometriosis [159,215,216,217]. Estrogens affect the microenvironment of the lower female reproductive system by increasing epithelial thickness, glycogen concentration, mucus secretion, promoting lactobacilli abundance and indirectly producing lactic acid [218]. Endometriosis, endometrial cancer and uterine fibroids are proliferative diseases associated with hormonal imbalance [219]. Estrogen promotes ectopic endometrial tissue formation and inflammatory activity in women, and endometriosis has been linked to changes in estrogen signaling [217]. For example, women with endometriosis show an increased pro-inflammatory and anti-apoptotic response to estradiol [220]. This may be due to changes in the expression of the nuclear estrogen receptor. Furthermore, elevated estrogen levels combined with the lack of opposing effects of progesterone create imbalances in progesterone and estrogen production, affecting the epithelium and potentially leading to uncontrolled profiling and chronic inflammation that promotes the development of endometriosis [221,222]. It is well known that the liver binds circulating estrogens to glucuronic acid, which does not bind to estrogen receptors (via glucuronidation). Glucuronic acid-conjugated estrogens are more hydrophilic, allowing them to be excreted in the bile (as bile salts) and then released into the stomach to eliminate conjugated toxins and hormones that are no longer needed [223] (Figure 3).

It has been proven that the intestinal microflora, and especially changes in the intestinal microflora (dysbiosis), are involved in the reactivation of estrogens through the bacterial secretion of the enzymatic β-glucuronidase, detected in some intestinal bacteria, such as *Escherichia coli*, *Bacteroides fragilis* and *Streptococcus agalactiae* [226], which are capable of deconjugating glucuronic acid [227,228]. As a result, active circulating estrogen is reabsorbed, which increases selectivity for beta estrogen receptor (ER) targets [229,230]. Many studies have linked ER variability to estradiol levels, although the mechanism is uncertain [229,231].

β-glucuronidase activity is essential for the formation of potentially harmful and carcinogenic metabolites in the intestines, as well as for the resorption of various chemicals in the circulatory system, such as estrogens [232]. β-glucuronidase promotes estrogen receptor binding, and activation of these receptors promotes proliferation. Changes in gut microbial diversity can disrupt or dysregulate circulating estrogen levels, fueling estrogen-mediated disease, contributing to hyper- or hypoestrogenic states [156,233].

Notably, the microbiota may be responsible for endometriosis by promoting inflammation and hormonal dysregulation (via the estrobolome), affecting cell proliferation/apoptosis, metabolism and oxidative stress, and increasing angiogenesis/vascularity [234,235,236,237,238]. As a result, research on the estrobolome is expected to provide new information that can be used in future interventions and therapies for endometriosis and other estrogen-mediated diseases. The gut microbiota plays a key role in the biotransformation of estrogen, which can affect hormonal balance and estrogen-related conditions such as endometriosis. Although research on the specific interactions between the estrobolome and the microbiome in endometriosis is still limited, there are several potential mechanisms for their interaction. This applies not only to the metabolism of estrogens in the body, but also to the development of inflammation or secondary metabolites. The gut microbiota can influence estrogen metabolism through the production of enzymes that can either increase or decrease estrogen levels. Some bacteria possess the ability to produce β-glucuronidase, an enzyme that can deconjugate estrogen metabolites, allowing them to be reabsorbed into circulation. This process can lead to increased exposure to estrogens and potentially exacerbate estrogen-driven conditions such as endometriosis [227,228]. The gut microbiota can produce metabolites that can influence estrogen metabolism and affect endometriosis. For example, certain bacterial species can produce SCFAs through the fermentation of dietary fibers. SCFAs can influence estrogen metabolism by modulating the expression of estrogen-metabolizing enzymes in the liver and other tissues. These metabolites can potentially impact estrogen levels and the hormonal balance involved in endometriosis [227]. 

## 5. Future Directions—The Role of Probiotics and Prebiotics in Endometriosis

The presence of bacteria in both the gut and uterus has been found to be significant in the development and progression of endometriosis. This understanding opens up potential avenues for improving endometriosis care. Modulating the microbiota, either through antibiotics or probiotics, could be a treatment and/or prevention of endometriosis. Probiotics and prebiotics are two components of the emerging field of microbiome-based therapies. While research specifically focused on the role of probiotics and prebiotics in endometriosis is limited, they have been studied in the context of other gynecological conditions and may have potential benefits for endometriosis [239].

Probiotics are live microorganisms that, when administered in adequate amounts, confer health benefits to the host. They are commonly found in certain foods or can be taken as supplements. Probiotics have been investigated for their potential effects on immune regulation, inflammation and gut dysbiosis, all of which are relevant in the context of endometriosis. While the specific strains and mechanisms of action are still being explored, probiotics may offer potential benefits like the modulation of immune response, restoration of gut microbiota and reduction of vaginal dysbiosis [240,241]. 

Probiotics can help regulate the immune system by promoting anti-inflammatory responses and balancing immune cell activity. This immune modulation could potentially reduce the inflammatory environment associated with endometriosis. Imbalances in the gut microbiota have been associated with various health conditions, including endometriosis. Probiotics may help restore microbial balance in the gut, potentially improving overall gut health and impacting systemic inflammation and immune responses. Some studies have explored the use of probiotics to restore a healthy vaginal microbiota, which could potentially be relevant for endometriosis-related symptoms such as pelvic pain and inflammation [142,240,241,242]. Studies have shown that oral administration of *Lactobacillus* in women with endometriosis can alleviate endometriosis-associated pain and reduce endometriotic lesions in mice. This effect may be achieved by increasing IL-12 concentration and enhancing NK cell activity, thus reversing immune dysregulation associated with endometriosis. Probiotic treatment with *Lactobacillus* has also been found to prevent the growth of endometriosis in rat models [243,244,245]. In order to increase the effectiveness of probiotics, it seems important to combine them with prebiotics. Prebiotics are indigestible fibers that serve as food for the beneficial bacteria in your gut. By selectively promoting the growth and activity of beneficial microbes, prebiotics can help improve the composition and function of the gut microbiota. While research focusing specifically on prebiotics in endometriosis is limited, their potential benefits for gut health and immune regulation are promising for future applications. Prebiotics primarily can stimulate the growth of beneficial bacteria, thereby increasing the diversity of microbes in the gut. This increased diversity may contribute to a healthier gut environment and potentially influence systemic inflammation and immune responses. Prebiotics may also support the integrity of the intestinal barrier by reducing the translocation of harmful bacteria and their products into the bloodstream. This can help reduce systemic inflammation and potentially affect symptoms associated with endometriosis [242,246].

## 6. Conclusions

Recently, the microbiological aspects of endometriosis have been investigated, but the nature of these interactions is still unclear. Our study gathers the latest knowledge about the involvement of microflora in endometriosis, mainly research on intestinal microflora with clinical results. According to this research, the development and progression of endometriosis may be closely related to the microbiome of the female reproductive system and intestines. This shows that monitoring microbiome diversity can be an effective marker in this clinically difficult disease. More research is needed to investigate the application of this fresh perspective on endometriosis, which may have a potential role in finding preventive, diagnostic and therapeutic options, and which will involve clinical trials. 

The results of the laboratory and clinical studies presented in this literature review confirm that the composition of the microbiome of patients with endometriosis differs from the composition of the microbiome of patients without this disease. The main problem with endometriosis is delayed diagnosis. Usually, the average time from onset of symptoms to diagnosis is about 8–10 years. The gold standard for diagnosis is laparoscopy, which has the disadvantage of being invasive. Identifying the composition of the disease-specific microbiome would enable the development of non-invasive diagnostics that could help reduce the incidence of delayed diagnoses, when the disease is usually at a more advanced stage. Endometriosis is a disease that greatly affects the quality of life of patients, so it is important to find new and effective forms of treatment for this disease. Understanding the functional role of microorganisms in endometriosis would also open the door to the use of antibiotics, prebiotics, probiotics and microbial transplants to alter the disease. However, to make this possible, more research is needed on the role of the microbiome in endometriosis.

## Figures and Tables

**Table 1:** Characteristics of selected proteins with biomarker molecule potential found in the urine of patients with endometriosis.

Gene	Name of Protein	Protein ID	Amino Acids	Mass (kDa)	Function	References
*ENG*	Endoglin	P17813	658	70.578	Vascular endothelium glycoprotein that plays an important role in the regulation of angiogenesis; Acts as TGF-beta coreceptor and is involved in the TGF-beta/BMP signaling cascade that ultimately leads to the activation of SMAD transcription factors; ENG was significantly higher in the ectopic endometriotic tissues than that in eutopic endometriotic tissue.	[98,99,100,101]
*LUM*	Lumican	P51884	338	38.429	Lumican demonstrated higher immunoreactivity and relative expression in the endometrium of women with PCOS compared to that of women with regular menstrual cycles.	[102,103]
*TGFB2*	Transforming Growth Factor Beta Receptor 2	P37173	567	64.568	Transmembrane serine/threonine kinase forming with the TGF-beta type I serine/threonine kinase receptor, TGFBR1, the non-promiscuous receptor for the TGF-beta cytokines TGFB1, TGFB2 and TGFB3.	[88,104]
*TSPAN1*	Tetraspanin-1	O60635	241	26.301	SPAN1 overexpression enhanced cell growth and invasion of ovarian clear cell carcinoma; TSPAN1 promoted endometriotic cell growth through AMPK activity.	[105,106]
*CD44*	CD44 Antigen	P16070	742	81.538	CD44 plays a role in cell–cell interactions, cell adhesion and migration, helping them to sense and respond to changes in the tissue microenvironment; The concentration of soluble CD44 in the serum and endometrial fluid of endometriosis patients was higher than in healthy women.	[107,108,109]
*TNC*	Tenascin	P24821	2201	240.853	Tenascin is a high-molecular-weight ECM protein and plays a critical role in tissue regeneration, hyperplastic and neoplastic processes; The modulation of tenascin as an extracellular matrix protein by E(2) in endometriotic stromal cells may be one of the factors playing a role in the development of endometriosis.	[110,111]
*CatG*	Cathepsin G	P08311	255	28.837	Serine protease with trypsin- and chymotrypsin-like specificity; The level of cathepsin G ascertained in endometrium tissue samples was over twice as high for the group of patients suffering from endometriosis as compared to the control group.	[112,113,114]
*DSP*	Desmoplakin	P15924	2871	331.774	Major high-molecular-weight protein of desmosomes; Regulates profibrotic gene expression in cardiomyocytes via activation of the MAPK14/p38 MAPK signaling cascade and increase in TGFB1 protein abundance.	[115,116]
*THBS1*	Thrombospondin 1	A8MZG1	94	10.053	Thrombospondin-1 serum levels correlate with pelvic pain in patients with ovarian endometriosis.	[117,118,119]
*PCDH1*	Protocadherin-1	Q08174	1060	114.743	Involved in cell–cell interaction processes and in cell adhesion; PCDHs mediate cell growth, cell cycle arrest, apoptosis and migration of endometrial cancer, underlining their critical roles in endometrial carcinogenesis.	[120,121]
*SPARCL1*	SPARC-like Protein 1	Q14515	664	75.208	SPARC enhanced fibronectin expression and promoted migration activity; SPARC was expressed in endometrial cancer tissues.	[122,123]
*AZGP1*	Zinc-alpha-2-glycoprotein	P25311	298	34.259	ZAG activity is controlled by hormones, the immune system and, interestingly, polyunsaturated fats and essential fatty acids; Elevated ZAG may not be the most specific biomarker for endometriosis, but it may point to other mechanisms potentially more diagnostic.	[124,125,126,127]
*ANXA2*	Annexin A2	P07355	339	38.604	Calcium-regulated membrane-binding protein whose affinity for calcium is greatly enhanced by anionic phospholipids; ANXA2 inhibition abrogated endometrial tissue growth, metastasis and angiogenesis in an adenomyosis nude mice model and significantly alleviated hyperalgesia.	[96,128]

## Data Availability

For more information, please contact the first author of this publication.
